# The impact on ambient air pollution and asthma-related admissions of COVID-19 transport restrictions: Caitriona Kelly

**DOI:** 10.1093/eurpub/ckac130.067

**Published:** 2022-10-25

**Authors:** C Kelly, P Kenny, M O'Dwyer, KI Quintyne

**Affiliations:** 1 Department of Public Health, North East, HSE, Kells, Ireland; 2 National Ambient Air Quality Unit, EPA, Dublin, Ireland; 3 School of Public Health, UCC, Cork, Ireland

## Abstract

Exposure to air pollution is a known risk factor for asthma exacerbations, emergency attendances and hospitalisations. In Europe, the main source of air pollution is the transport industry, and so the COVID-19 transport restrictions provided an opportunity to examine if reduction in traffic had a demonstrable impact on ambient air quality and asthma-related admissions. Routinely collected data was used to conduct a retrospective population cohort study. The Environmental Protection Agency provided daily nitrogen dioxide (NO2) and particulate matter (PM) concentrations for Dublin, and all asthma-related admissions were collected from the Hospital In-Patient Enquiry system. The two years prior to the pandemic were compared with the period of transport restrictions (from March 2020). During the period of restrictions, there was a significant reduction in the mean number of daily asthma admissions (2.8 v 4.5 admissions p < 0.001). There was also a significant decrease in mean daily concentrations in two pollutants: NO2 (16.7 v 24.0µg/m3 p < 0.001) and PM2.5 (7.8 v 8.9µg/m3 p = 0.002). Only NO2 had a statistically significant correlation with asthma admissions (r = 0.132 p < 0.001). Transport restrictions introduced to mitigate against COVID-19 led to improvements in air quality, as seen by the reductions in pollutant concentrations. Previously described associations between pollutants and asthma, would suggest that these improvements in air quality contributed to the reduction in asthma admissions. Whereas the primary source of NO2 is transport emissions, PM is made up of particles from multiple sources, which likely explains the lack of correlation between asthma admissions and PM. Public Health need to advocate for transport policies which can improve air quality, and as a result, public health.

**Key messages:**

- Poor air quality poses a significant health challenge requiring public health input and advocacy.
- Reducing vehicle traffic can improve air quality which would have a positive impact on public health.

